# Stagnation arising through intermittent usage is associated with increased viable but non culturable *Legionella* and amoeba hosts in a hospital water system

**DOI:** 10.3389/fcimb.2023.1190631

**Published:** 2023-06-07

**Authors:** Muhammad Atif Nisar, Kirstin E. Ros, Melissa H. Brown, Richard Bentham, Giles Best, James Xi, Jason Hinds, Harriet Whiley

**Affiliations:** ^1^College of Science and Engineering, Flinders University, Bedford Park, SA, Australia; ^2^Australian Research Council Training Centre for Biofilm Research and Innovation, Flinders University, Bedford Park, SA, Australia; ^3^College of Medicine and Public Health, Flinders University, Bedford Park, SA, Australia; ^4^Flow Cytometry Facility, Flinders University, Bedford Park, SA, Australia; ^5^Enware Australia Pty Ltd., Caringbah, NSW, Australia

**Keywords:** Legionnaires’ disease, water safety plan, building plumbing systems, free-living amoebae, potable water

## Abstract

Hospital water systems are a significant source of *Legionella*, resulting in the potentially fatal Legionnaires’ disease. One of the biggest challenges for *Legionella* management within these systems is that under unfavorable conditions *Legionella* transforms itself into a viable but non culturable (VBNC) state that cannot be detected using the standard methods. This study used a novel method (flow cytometry-cell sorting and qPCR [VFC+qPCR] assay) concurrently with the standard detection methods to examine the effect of temporary water stagnation, on *Legionella* spp. and microbial communities present in a hospital water system. Water samples were also analyzed for amoebae using culture and *Vermamoeba vermiformis* and *Acanthamoeba* specific qPCR. The water temperature, number and duration of water flow events for the hand basins and showers sampled was measured using the Enware Smart Flow^®^ monitoring system. qPCR analysis demonstrated that 21.8% samples were positive for *Legionella* spp., 21% for *L. pneumophila*, 40.9% for *V. vermiformis* and 4.2% for *Acanthamoeba*. All samples that were *Legionella* spp. positive using qPCR (22%) were also positive for VBNC *Legionella* spp.*;* however, only 2.5% of samples were positive for culturable *Legionella* spp. 18.1% of the samples were positive for free-living amoebae (FLA) using culture. All samples positive for *Legionella* spp. were also positive for FLA. Samples with a high heterotrophic plate count (HPC ≥ 5 × 10^3^ CFU/L) were also significantly associated with high concentrations of *Legionella* spp. DNA, VBNC *Legionella* spp./*L. pneumophila* (*p* < 0.01) and *V. vermiformis* (*p* < 0.05). Temporary water stagnation arising through intermittent usage (< 2 hours of usage per month) significantly (*p* < 0.01) increased the amount of *Legionella* spp. DNA, VBNC *Legionella* spp./*L. pneumophila*, and *V. vermiformis;* however, it did not significantly impact the HPC load. In contrast to stagnation, no relationship was observed between the microbes and water temperature. In conclusion, *Legionella* spp. (DNA and VBNC) was associated with *V. vermiformis*, heterotrophic bacteria, and stagnation occurring through intermittent usage. This is the first study to monitor VBNC *Legionella* spp. within a hospital water system. The high percentage of false negative *Legionella* spp. results provided by the culture method supports the use of either qPCR or VFC+qPCR to monitor *Legionella* spp. contamination within hospital water systems.

## 1 Introduction

*Legionella* is an opportunistic premise plumbing pathogen and etiological agent of Legionnaires’ disease (LD), a potentially fatal pneumonia like infection (Cunha et al., 2016). *Legionella* is ubiquitous in natural and engineered water systems and transmitted through aspiration or inhalation of *Legionella* contaminated water or aerosols (Schwake et al., 2021). Globally the incidence of LD has been increasing. In 2021, the US Centers for Disease Control and Prevention (CDC) reported 8260 confirmed cases of LD in USA (Centers for Disease Control and Prevention, 2022). In Australia, 524 confirmed cases of legionellosis were reported in 2020 (Australian Government Department of Health and Aged Care, 2021). According to the European Centre for Disease Prevention and Control (ECDC) 11,298 confirmed cases of LD were documented across European countries in 2019. However, in 2020 the number decreased to 8,372; this reduction may be associated with COVID-19 pandemic lockdown restrictions or a decrease in focus on LD. In 2021, 10,723 confirmed cases of LD were documented of which 5.4% were nosocomial infections (The European Legionnaires’ Disease Surveillance Network, 2022). The actual number of legionellosis cases is understated, because in the majority of cases Pontiac fever remains unnoticed and the etiological agent of pneumonia remains unrecognized (Cassell et al., 2019). There are at least 60 distinct species of *Legionella*, with *L. pneumophila* sg.1 being the most common cause of outbreaks (Khodr et al., 2016; Miyashita et al., 2020). Initially, cooling towers were considered to be the main source of *Legionella* spp., but subsequent investigations have identified that engineered water systems are a major source of LD (Kanarek et al., 2022). Those at greatest risk of infection are the elderly and immunocompromised individuals, and as such nosocomial outbreaks associated with hospital engineered water systems are of significant concern (Bartram et al., 2007).

A range of factors influence the survival and persistence of *Legionella* spp. in hospital water systems including: biofilms, nutrients, disinfectants, protozoa hosts, water temperature, flow dynamics and stagnation (Abdel-Nour et al., 2013; Whiley et al., 2017; Nisar et al., 2020b). Naturally, *Legionella* spp. infects and survives within a wide range of polyphyletic protozoan hosts, with *Acanthamoeba* and *Vermamoeba* the most commonly identified hosts in potable water (Boamah et al., 2017; Best and Abu Kwaik, 2018; Nisar et al., 2020a). Intracytoplasmic *Legionella* spp. are protected from adverse environmental conditions (Best and Abu Kwaik, 2018), with *Legionella* spp. released from host protozoa more virulent and pathogenic in nature (Fields et al., 2002; Boamah et al., 2017). Additionally, *Legionella* spp. intrinsically tolerate water disinfection treatments by entering into a metabolically inactive but highly resistant and potentially pathogenic “viable but non-culturable” (VBNC) state (Kirschner, 2016). Under suitable environmental conditions, and in the presence of protozoa hosts, VBNC *Legionella* spp. can resuscitate back into a culturable state (Dietersdorfer et al., 2018). VBNC *Legionella* spp. are a significant challenge to water quality management as they cannot be detected using the standard culture-based method (International Organization for Standardization, 2017; Standards Australia, 2017). *Legionella* spp. specific quantitative PCR (qPCR) assay is an alternative method typically used to detect the genomic load of *Legionella* spp. (International Organization for Standardization, 2019); however, it cannot distinguish between culturable, dead and VBNC *Legionella* spp. (Kirschner, 2016). As such, there are currently limited studies that investigate the survival of VBNC *Legionella* spp. in engineered water systems.

Water stagnation in engineered water systems is categorized into two different types; permanent, and temporary stagnation (Peter and Routledge, 2018; Nisar et al., 2020b). Permanent stagnation is complete stagnation of plumbing structures, such as dead-ends and dead-legs (Nisar et al., 2020b). However, in engineered water systems, water can also stagnate in storage tanks, plumbing piping network, and within components at the water outlets for a few hours to weeks and even months (Bartram et al., 2007; Manuel et al., 2009). This type of water stagnation is known as intermittent or temporary stagnation (Manuel et al., 2009; Peter and Routledge, 2018). The relationship between *Legionella* spp. and permanent stagnation is well characterized (Totaro et al., 2018; Nisar et al., 2020b). However, less is known about the relationship between *Legionella* spp. and temporary stagnation. Therefore, this study examined the role of temporary stagnation arising through intermittent water usage on the persistence of *Legionella* spp. and free-living amoebae (FLA) within a hospital water system.

This study was the first study to utilize a novel method to enumerate VBNC *Legionella* spp. and *L. pneumophila* from environmental water samples and investigate relationships with protozoan hosts. This study utilized the Enware Smart^®^ Flow monitoring system to examine the relationships between water flow (arising through water outlet usage) and temperature with *Legionella* spp., *L. pneumophila* and amoeba hosts. The specific aims of this study were as follows, to: (1) determine the prevalence of *Legionella* spp./*L. pneumophila* and FLA in a hospital water system; (2) examine the relationship between *Legionella* spp. and potential protozoan hosts; and (3) monitor the effect of sampling phases (months), water temperature, flow dynamics and stagnation on persistence of *Legionella* spp. in the hospital water system. To our knowledge, this is the first comprehensive study that has quantified VBNC *Legionella* spp. and FLA from a hospital water system under dynamic flow and temperature conditions.

## 2 Materials and methods

### 2.1 Sample collection and processing

From March 2021 to June 2022, water (n = 120) and biofilm (n = 46) samples were collected from the engineered water system of an Australian hospital located in New South Wales, Australia. The sampling was done in different phases, where the categorization was: March 2021 as phase 1, April 2021 as phase 2, November 2021 as phase 3 and June 2022 as phase 4. All water and biofilm samples were collected, transported and stored as recommended by standard guidelines (International Organization for Standardization, 2018; Centers for Disease Control and Prevention, 2019). For the water samples, 1 L first flush hand basin or shower water was collected in sterile wide-mouth screw capped plastic bottles (2105-0032, Nalgene™). For the biofilm samples, visible biofilm was scraped from the inside of tap faucet or shower head using sterile polyurethane-tipped swabs (CleanFoam^®^ TX751B, Texwipe^®^), then 5 to 10 mL of water was added and placed with the swab in a sterile screw capped tube. For both the water and biofilm samples, 0.5 mL 0.1 N sodium thiosulfate (124270010, ACROS Organics™) was added to neutralize pre-existing chlorine-based chemical disinfectants. All samples were transported and kept at 5 ± 2°C and processed within 72 hours. The samples were vacuum filtered through 47 mm diameter 0.22 µm polycarbonate membrane (GTTP04700, Isopore™). The filtered residues were resuspended in 3 mL sterile distilled water. This sample suspension was used for further microbiological and molecular testing.

### 2.2 Water flow and temperature data

Parameters related to water temperature and flow dynamics were monitored in the hospital water system using the Enware Smart^®^ Flow monitoring system. Briefly, this monitoring system measures water system delivery temperatures using temperature probes located at the hot water inlet, cold water inlet, and outlet of the thermostatic mixing valves (TMV) and the hot water inlet and cold water inlet of hand basin faucets. Water flow was measured using flow switches located at the hot water inlet and cold water inlet of both the TMVs and hand basin faucets (Whiley et al., 2019). The temperature data of the hot water supply, cold water supply and outlet was collected for the entire duration of the sampling period. For analysis these measurements were separated into a period one week and one month prior to a water sampling event. In terms of flow regime, the total duration (hours) and number (counts) of flushing events for a period of one week and one month prior to sampling were recorded. The total duration (hours) of flushing events were divided into low and high flow regimes with categorization as: low flow regime; 0 to < 2 hours per month, and high flow regime; ≥ 2 to 40 hours per month.

### 2.3 Molecular analysis

Quantification of *Legionella* spp. (16 rDNA gene) and *L. pneumophila* (*mip* gene) was performed using ISO/TS12869:2019 quantitative polymerase chain reaction (qPCR) assays (International Organization for Standardization, 2019). The 18S rDNA gene was amplified for the quantification of *Acanthamoeba* and *Vermamoeba vermiformis* (Qvarnstrom et al., 2006; Scheikl et al., 2016). *Legionella* spp. (GenBank Acc CP021281), *L. pneumophila* (GenBank Acc KR902705), *Acanthamoeba castellanii* (GenBank Acc U07413) and *V. vermiformis* (GenBank Acc KT185625) gBlocks gene fragments (IDT™) were used as a positive control and for the preparation of a standard curve using ten-fold serial dilutions. Using the Aquadien™ kit (3578121, BIO-RAD Laboratories Ltd.), genomic DNA was extracted from each water and biofilm sample before being subjected to a qPCR assay. The qPCR reaction mixture consisted of microbe-specific primers (Bio-Rad Laboratories Ltd.), 1X PCR reaction buffer (2X SsoAdvanced™ universal probes supermix:172-5281, Bio-Rad Laboratories Ltd.), and DNA template. To detect the potential presence of environmental inhibitors of the qPCR assays, both the purified and a one in ten dilution of extracted DNA was used as template (Hayes-Phillips et al., 2019; Nisar et al., 2022). Using a Rotor-Gene Q thermal cycler (Qiagen Ltd.), each template DNA was subjected to the qPCR assay in triplicate (Nisar et al., 2022). All fluorescence labelled probes and primers used in this study are presented in **Table S1** (**Supplementary Material**).

### 2.4 Microbiological analysis

Isolation of culturable *Legionella* spp. and *L. pneumophila* was performed in accordance with the standard guidelines (International Organization for Standardization, 2017; Standards Australia, 2017). Briefly, samples were heat treated (50 ± 1°C for 30 ± 2 minutes) and/or acid treated (HCl-KCl buffer treatment for 5 ± 0.5 minutes) to reduce the contamination of interfering microbes. An aliquot of treated sample was then spread on *Legionella* agar (CM1203, Oxoid Ltd.) supplemented with GVPC (glycine, vancomycin, polymyxin B and cycloheximide: SR0152, Oxoid Ltd.) and *Legionella* growth supplement (α-ketoglutarate, buffer/potassium hydroxide, ferric pyrophosphate, and _L_-cysteine: SR0110C, Oxoid Ltd.). The inoculated plates were incubated at 37 ± 1°C for 7 days and examined every day. Suspected *Legionella-*like colonies were counted from each plate and evaluated by *Legionella* latex agglutination test kit (DR0800, Oxoid Ltd.). This kit identifies genus *Legionella* and further characterizes various species and serogroups with overall 99% sensitivity and 100% specificity. Furthermore, all *Legionella-*like colonies were confirmed through *Legionella* spp. specific qPCR assays. To determine the heterotrophic plate counts (HPC), an aliquot from each sample was inoculated on R_2_A agar (CM0906, Oxoid Ltd.) and incubated at 35 ± 1°C. The colonies were counted after 2, 5 and 7 days of incubation. The results for *Legionella* spp. and heterotrophic bacteria were expressed in colony forming units (CFU)/L for water samples and CFU for the biofilm samples. Isolation of culturable FLA was performed by inoculating an aliquot of each sample on heat-inactivated (57°C for 45 minutes) *Escherichia coli* American Type Culture Collection 700891™ supplemented 1.5% non-nutrient agar (Eco-NNA: CM0003, Oxoid Ltd.) (Nisar et al., 2022). The plates were incubated at 25 ± 1°C for 14 days and amoebal growth was examined daily using an inverted light microscope (AMEFC4300, EVOS™ FL, Thermo Fisher Scientific). All monoxenic amoebae cultures were characterized by microscopic examination and sequence analysis of 18S rDNA gene.

### 2.5 Quantification of VBNC *Legionella* spp. and *L. pneumophila*

VBNC *Legionella* spp. and *L. pneumophila* were detected and quantified by flow cytometry-cell sorting and qPCR (VFC+qPCR) assay (Nisar et al., 2023). Briefly, 300 μL sample suspension was resuspended in 200 µL of filter sterilized staining buffer (0.01% Tween-20 and 1 mM EDTA in 1X PBS, pH 7.4 ± 0.1), followed by addition of 48 µM propidium iodide (PI) and 420 nM thiazole orange (TO) dyes (cell viability kit Cat # 349480, Becton Dickinson, Franklin Lakes, USA). The mixture was incubated at 5°C for 15 minutes. Then, using a FACS Aria Fusion instrument, (Becton Dickinson, Franklin Lakes, USA) cells were analyzed and segregated into dead (PI/TO), alive (potentially culturable: TO), and injured (potentially VBNC: PI/TO) cell populations. From each sample, the injured cell fraction was isolated and subjected to DNA extraction and quantification of *Legionella* spp. and *L. pneumophila* gene markers (Nisar et al., 2023).

### 2.6 Data analysis

The data are described in logarithmic form with base 10 (log_10_). The percentage of both bacterial and amoebae isolates was determined based on phases or contamination levels and plotted in Microsoft^®^ Excel^®^. Statistical calculations were made using R studio (version 4.2.2) and graphically presented by using “ggplot2 (version 3.3.6)” package (Wickham, 2016). Briefly, the Shapiro-Wilk test was used to assess normality of analyzed quantitative parameters. For comparison of the means of the quantitative parameters (i.e., either GU or CFU of *Legionella* spp./*L. pneumophila*, HPC, *Acanthamoeba* and *V. vermiformis*), a non-parametric Kruskal-Wallis test was used. Finally, the non-parametric Spearman’s correlation (ρ) test was used to evaluate relationships among different variables (i.e., *Legionella* spp./*L. pneumophila*, HPC, *Acanthamoeba* and *V. vermiformis*). A statistically significant difference among the quantitative parameters was defined by *p* values of less than 0.05.

## 3 Results

### 3.1 Occurrence of *Legionella* spp. and *L. pneumophila*

**Figure 1:**
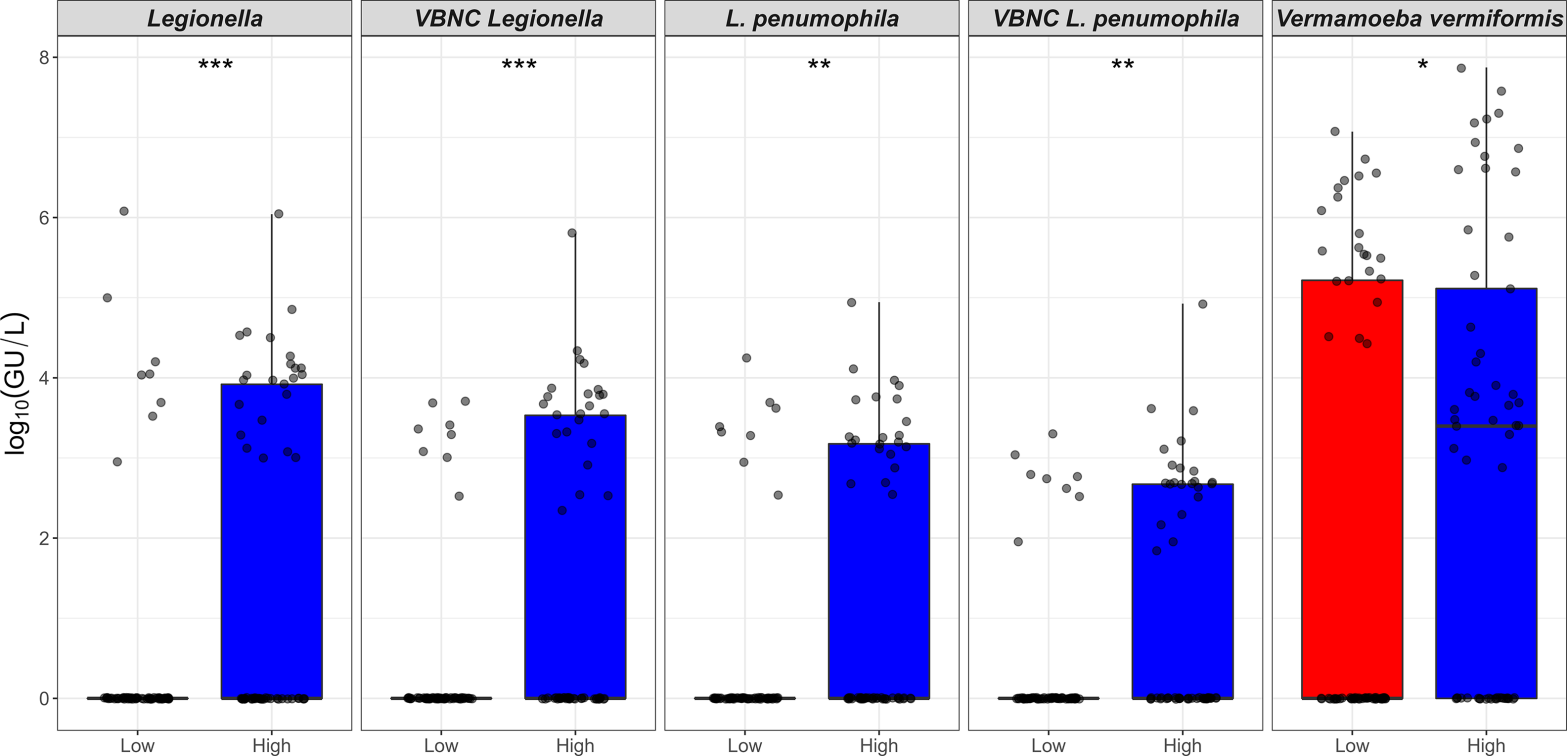
Prevalence (%) of *Legionella* spp. and free-living amoeba in a hospital water system. A total of 166 water (hand basin and shower) and biofilm (tap faucet) samples were collected in March 2021 (Phase 1), April 2021 (Phase 2), November 2021 (Phase 3), and June 2022 (Phase 4). Total amounts of *Legionella* spp./*L. pneumophila*, *Acanthamoeba*, and *Vermamoeba vermiformis* were detected and quantified by qPCR assays. Culturable *Legionella* spp. and amoebae were detected by standard microbiological culturing procedures. VBNC *Legionella* spp. and VBNC *L. pneumophila* were detected and quantified by flow cytometry-cell sorting and qPCR assay. The p values are: *p ≤ 0.05, **p ≤ 0.01, and ***p ≤ 0.001.

**Table 1** presents an overview of the percentage of samples identified as positive for *Legionella* spp. and FLA using the different detection methods. All samples that were qPCR positive for *Legionella* spp. were also positive for FLA and VBNC *Legionella.* Specifically, 21.7% (n = 36/166) of total samples were positive for *Legionella* spp. DNA (16S rDNA gene) with a concentration range of 9 × 10^2^ to 1.5 × 10^6^ GU (**Tables 1**, **2**). *L. pneumophila* DNA (*mip* gene) was present in 21% samples (n = 35/166) with a concentration ranging from 3.5 × 10^2^ to 9 × 10^4^ GU (**Tables 1**, **2**). All *L. pneumophila* positive samples were also positive for *Legionella* spp. During phase 1, 58.06% (n = 18/31) of the samples tested positive for *Legionella* spp. DNA, whereas in the 2^nd^ phase 7.31% (n = 3/41), and 4^th^ phase 28.84% (n = 15/52) of the collected samples tested positive for *Legionella* spp. DNA (**Figure 1**). However, in phase 3 none of the samples were positive for either *Legionella* spp. or *L. pneumophila* DNA. Standard culturing demonstrated that only four samples (two in phase 1 and two in phase 4) were positive for culturable *Legionella* spp., which were identified as non-*pneumophila Legionella* using serology and qPCR. The VFC+qPCR assay demonstrated that all samples positive for either *Legionella* spp. or *L. pneumophila* DNA (according to the qPCR assay) also contained VBNC cells (**Figure 1**; **Table 1**). Therefore, of the 36 samples that were positive for VBNC *Legionella* spp., the standard microbiological culturing assay returned a false negative result for 32 of them (88.9%). For analysis, the VBNC *Legionella* spp. and *L. pneumophil*a samples were categorized into three groups based on concentration i.e., low (< 10^3^ GU/L), medium (≥ 10^3^ to 10^4^ GU/L) and high (> 10^4^ GU/L) contamination. Based on this grouping it was found that in phase 1, 14.3% (n = 4/31) of the water samples were positive for high VBNC *Legionella* spp. contamination, whereas in the 4^th^ phase 33.3% (n = 10/52) water samples were positive for high VBNC *Legionella* spp. contamination (**Supplementary Material, Figure S1A**). It was found that the lower level of VBNC *L. pneumophila* occurred more frequently in the samples collected during phase 1 (53.5%, n = 15/31) and 4 (23.3%, n = 7/52). Only 6.7% (phase 4: n = 2/52) water samples contained high levels of VBNC *L. pneumophil*a contamination (**Supplementary Material, Figure S1B**). Based on sampling sites it was found that in hand basin water, 13.4% (n = 9/67) samples were positive for high VBNC *Legionella* spp. contamination, whereas in shower water, 9.4% (n = 5/53) samples were positive for high VBNC *Legionella* spp. contamination (**Supplementary Material, Figure S2A**). However, the majority of hand basin (22.4%, n = 15/67) and shower (15.1%, n = 8/53) water samples contained low levels of VBNC *L. pneumophila* contamination (**Supplementary Material, Figure S2B**). Overall, both qPCR and VFC+qPCR assays clearly demonstrated that the standard culturing assay is frequently unable to detect *Legionella* spp./*L. pneumophila* present in the hospital water system.

**Table 1:** Prevalence of *Legionella* spp., *Vermamoeba vermiformis*, *Acanthamoeba* and total free-living amoeba in a hospital water system using different detection methods.

Sample (n)	*Number of Legionella* positive samples (%)					Number of free-living amoeba positive samples (%)		
	qPCR assay		VFC+qPCR		Culture assay	qPCR assay		Culture assay
	*Legionella*	*L. pneumophila*	*Legionella*	*L. pneumophila*		*Acanthamoeba*	*V. vermiformis*	
Sampling phase 1 (March 2021)								
Hand basin water (n = 16)	11	11	11	11	1	3	15	9
Shower water (n = 12)	4	4	4	4	1	0	9	6
Tap faucet biofilm (n = 3)	3	3	3	3	0	1	2	1
**Total (n = 31)**	**18 (58.06%)**	**18 (58.06%)**	**18 (58.06%)**	**18 (58.06%)**	**2 (6.45%)**	**4 (12.9%)**	**26 (83.87%)**	**16 (51.61%)**
Sampling phase 2 (April 2021)								
Hand basin water (n = 17)	2	2	2	2	0	0	11	8
Shower water (n = 13)	1	1	1	1	0	0	4	2
Tap faucet biofilm (n = 11)	0	0	0	0	0	0	5	0
**Total (n = 41)**	**3 (7.31%)**	**3 (7.31%)**	**3 (7.31%)**	**3 (7.31%)**	**0**	**0**	**20 (48.78%)**	**10 (24.39%)**
Sampling phase 3 (November 2021)								
Hand basin water (n = 18)	0	0	0	0	0	1	0	1
Shower water (n = 14)	0	0	0	0	0	0	0	0
Tap faucet biofilm (n = 10)	0	0	0	0	0	0	1	1
**Total (n = 42)**	**0**	**0**	**0**	**0**	**0**	**1 (2.38%)**	**1 (2.38%)**	**2 (4.76%)**
Sampling phase 4 (June 2022)								
Hand basin water (n = 16)	8	7	8	7	0	1	10	0
Shower water (n = 14)	5	5	5	5	0	0	7	0
Tap faucet biofilm (n = 22)	2	2	2	2	2	1	4	2
**Total (n = 52)**	**15 (28.84%)**	**14 (26.92%)**	**15 (28.84%)**	**14 (26.92%)**	**2 (3.84%)**	**2 (3.84%)**	**21 (48.38%)**	**2 (3.84%)**

**Table 2:** The minimum and maximum microbial concentrations present in the positive water and biofilm samples.

Microbes	Minimum concentration	Maximum concentration
Hand basin water (n = 67)		
*Legionella* DNA (GU/L)	1 × 10^3^	1.5 × 10^6^
VBNC *Legionella* (GU/L)	2.5 × 10^2^	6.5 × 10^5^
*L. pneumophila* DNA (GU/L)	3.5 × 10^2^	9 × 10^4^
VBNC *L. pneumophila* (GU/L)	6 × 10^2^	8.5 × 10^4^
*Vermamoeba vermiformis* (GU/L)	7.5 × 10^2^	7.5 × 10^7^
*Acanthamoeba* (GU/L)	1 × 10^3^	5 × 10^3^
Heterotrophic plate count (CFU/L)	10	1.5 × 10^5^
Shower water (n = 53)		
*Legionella* DNA (GU/L)	9 × 10^2^	7 × 10^4^
VBNC *Legionella* (GU/L)	3.5 × 10^2^	2.5 × 10^4^
*L. pneumophila* DNA (GU/L)	3.5 × 10^2^	9.5 × 10^3^
VBNC *L. pneumophila* (GU/L)	70	4.5 × 10^3^
*Vermamoeba vermiformis* (GU/L)	1 × 10^3^	4 × 10^7^
*Acanthamoeba* (GU/L)	0	0
Heterotrophic plate count (CFU/L)	10	1.5 × 10^5^
Tap faucet biofilm (n = 46)		
*Legionella* DNA (GU)	1 × 10^4^	3.5 × 10^5^
VBNC *Legionella* (GU)	1.5 × 10^2^	3 × 10^4^
*L. pneumophila* DNA (GU)	7.5 × 10^2^	1.5 × 10^4^
VBNC *L. pneumophila* (GU)	1 × 10^2^	1 × 10^4^
*Vermamoeba vermiformis* (GU)	1 × 10^3^	1 × 10^6^
*Acanthamoeba* (GU)	4.5 × 10^3^	8 × 10^3^
Heterotrophic plate count (CFU)	15	7.5 × 10^4^

### 3.2 Occurrence of heterotrophic bacteria and FLA

Both the hand basin and shower water samples contained HPC counts ranging from 10 to 1.5 × 10^5^ CFU/L (**Table 2**). In the biofilm samples the HPC load ranged from 15 to 7.5 × 10^4^ CFU/sample (**Table 2**). In case of FLA, the *V. vermiformis* gene marker was present in 40.9% (n = 68/166) of samples with concentrations ranging from 7.5 × 10^2^ to 7.5 × 10^7^ GU (**Tables 1**, **2**). The *Acanthamoeba* gene marker was detected only in hand basin water (3.01%, n = 5/166) and biofilm samples (1.2%, n = 2/166) with a range of 1 × 10^3^ to 8 × 10^3^ GU (**Tables 1**, **2**). Culturable amoebae were identified in 18.1% (n = 30/166) samples; however, due to fungal overgrowth 13 isolates were unable to develop monoxenic cultures, of these 13, five isolates showed acrasid amoebae-like morphology. Only 17 isolates developed monoxenic cultures which were further characterized on the basis of cellular morphology and sequence analysis of 18S rDNA gene. Light microscopy revealed that isolates harbored monotactic morphotype and developed spherical cysts consisting of distinct inner and outer walls. Based on 18S rDNA sequencing, all these monoxenic isolates were identified as *V. vermiformis*.

### 3.3 Relationship among *Legionella* spp., HPC, and FLA

**Figure 2:**
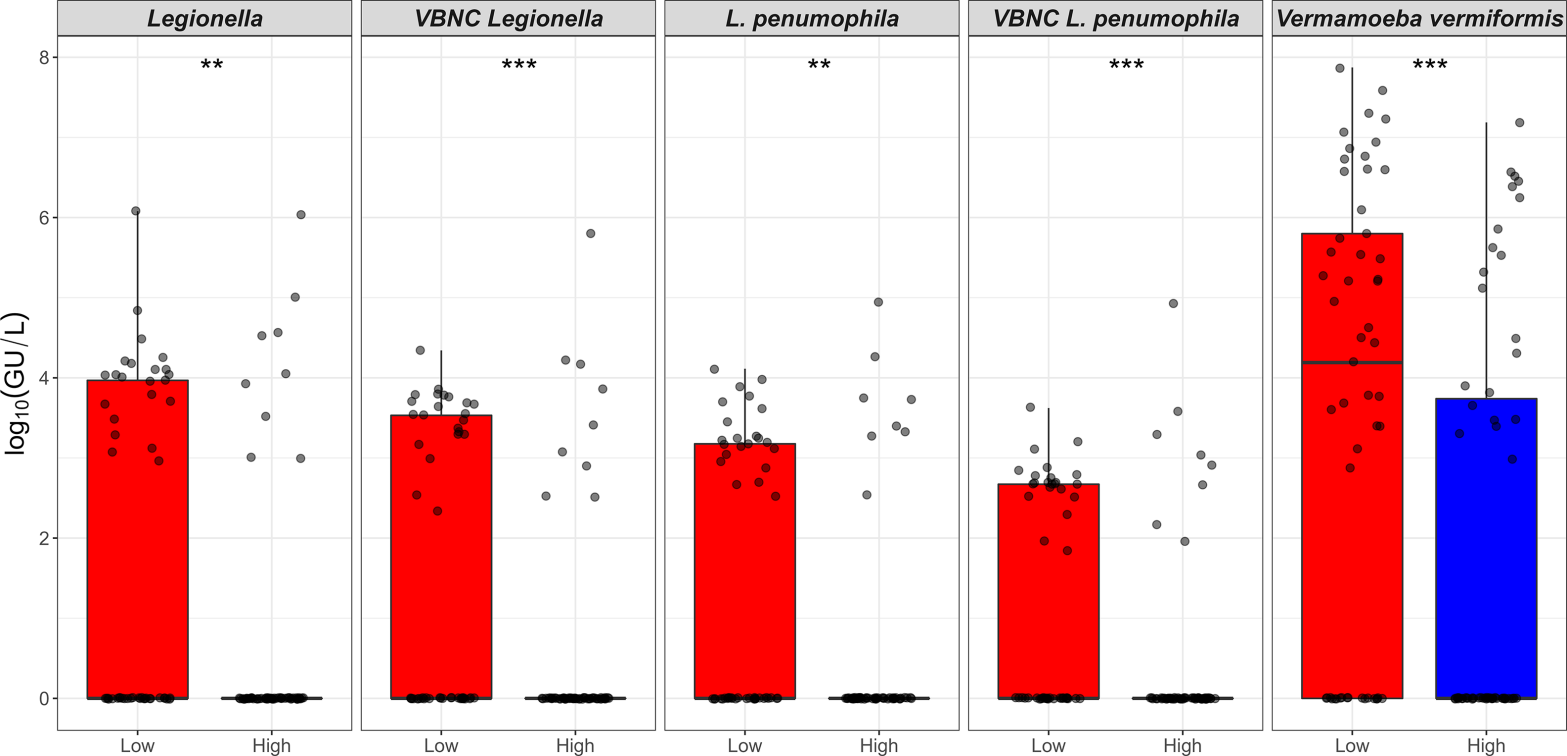
Relationship between the heterotrophic plate count and *Legionella* spp./*Vermamoeba vermiformis*. X-axis represents HPC level that is categorised into low (10 to < 5 × 10^3^ CFU/L) and high (≥ 5 × 10^3^ to 1.5 × 10^5^ CFU/L) contamination. Y-axis represents log_10_(GU/L) of *Legionella* spp., VBNC *Legionella* spp., *L. pneumophila*, VBNC *L. pneumophila*, and *V. vermiformis*. The p values are: **p ≤ 0.01, and ***p ≤ 0.001.

All shower and hand basin water samples were classified into two groups based on the HPC levels i.e., low (10 to < 5 × 10^3^ CFU/L) and high (≥ 5 × 10^3^ CFU/L) contamination. Kruskal-Wallis analysis demonstrated that quantity of both *Legionella* spp. DNA and VBNC *Legionella* spp. were significantly (*p* < 0.001) higher in water samples with high levels of HPC load (**Table 3**; **Figure 2**). Similarly, water samples having greater levels of HPC load harbored significantly higher concentrations of *L. pneumophila* DNA (Kruskal-Wallis test, *p* < 0.01), VBNC *L. pneumophila* (Kruskal-Wallis test, *p* < 0.01), and *V. vermiformis* (Kruskal-Wallis test, *p* < 0.05) (**Tables 3**; **Figure 2**). Furthermore, all samples characterized as positive for *Legionella* spp./*L. pneumophila* (DNA, culturable, and VBNC cells) were also positive for either the *V. vermiformis* gene marker or culturable amoebae. Furthermore, Spearman’s analysis demonstrated both *Legionella* spp./*L. pneumophila* DNA and *Legionella* spp./*L. pneumophila* VBNC cells were positively correlated (*p* < 0.001) with *V. vermiformis* (**Table 4**). Overall, these results suggested that in hospital water system, high levels of HPC load and *V. vermiformis* are positively associated with both *Legionella* spp./*L. pneumophila* DNA and VBNC cells.

**Table 3:** Effect of flow regime (flushing duration one-month prior to sampling) and heterotrophic plate counts of hand basin and shower water microbes.

Microbes in hand basin and shower water	Flow regime (one-month prior sampling) *		Heterotrophic plate count **	
	Low (0 to < 2 hours/month)	High (≥ 2 to 40 hours/month)	Low (10 to < 5 × 10^3^ CFU/L)	High (≥ 5 × 10^3^ to 1.5 × 10^5^ CFU/L)
**Total *Legionella* (GU/L)**	1.783 ± 2.043	0.637 ± 1.559	0.532 ± 1.443	1.604 ± 2.015
**VBNC *Legionella* (GU/L)**	1.560 ± 1.771	0.551 ± 1.366	0.413 ± 1.100	1.455 ± 1.839
**Total *L. pneumophila* (GU/L)**	1.472 ± 1.671	0.495 ± 1.284	0.429 ± 1.148	1.326 ± 1.698
**VBNC *L. pneumophila* (GU/L)**	1.206 ± 1.372	0.416 ± 1.107	0.344 ± 0.921	1.106 ± 1.442
***Vermamoeba vermiformis* (GU/L)**	3.578 ± 2.835	1.755 ± 2.508	1.983 ± 2.763	2.952 ± 2.755
**Heterotrophic plate count (CFU/L)**	3.742 ± 1.010	3.656 ± 0.855	–	–

Data is log transformed and shown as mean ± standard deviation.

*Microbial loads (except heterotrophic plate count) in the low flow regime are significantly higher than in the high flow regimes (Kruskal-Wallis analysis, p < 0.05).

**Microbial loads in high heterotrophic plate counts are significantly higher than low heterotrophic plate counts (Kruskal-Wallis analysis, p < 0.05).

**Table 4:** Correlation between *Legionella* spp. and *Vermamoeba vermiformis* in hand basin and shower water samples.

	Spearman’s rank correlation			
	*Legionella* DNA	VBNC *Legionella*	*L. pneumophila* DNA	VBNC *L. pneumophila*
***Vermamoeba vermiformis***	ρ = 0.5819 *p* < 0.001	ρ = 0.5833 *p* < 0.001	ρ = 0.5955 *p* < 0.001	ρ = 0.5826 *p* < 0.001

### 3.4 Influence of flow regimes and on *Legionella* spp., HPC, and FLA

**Figure 3:**
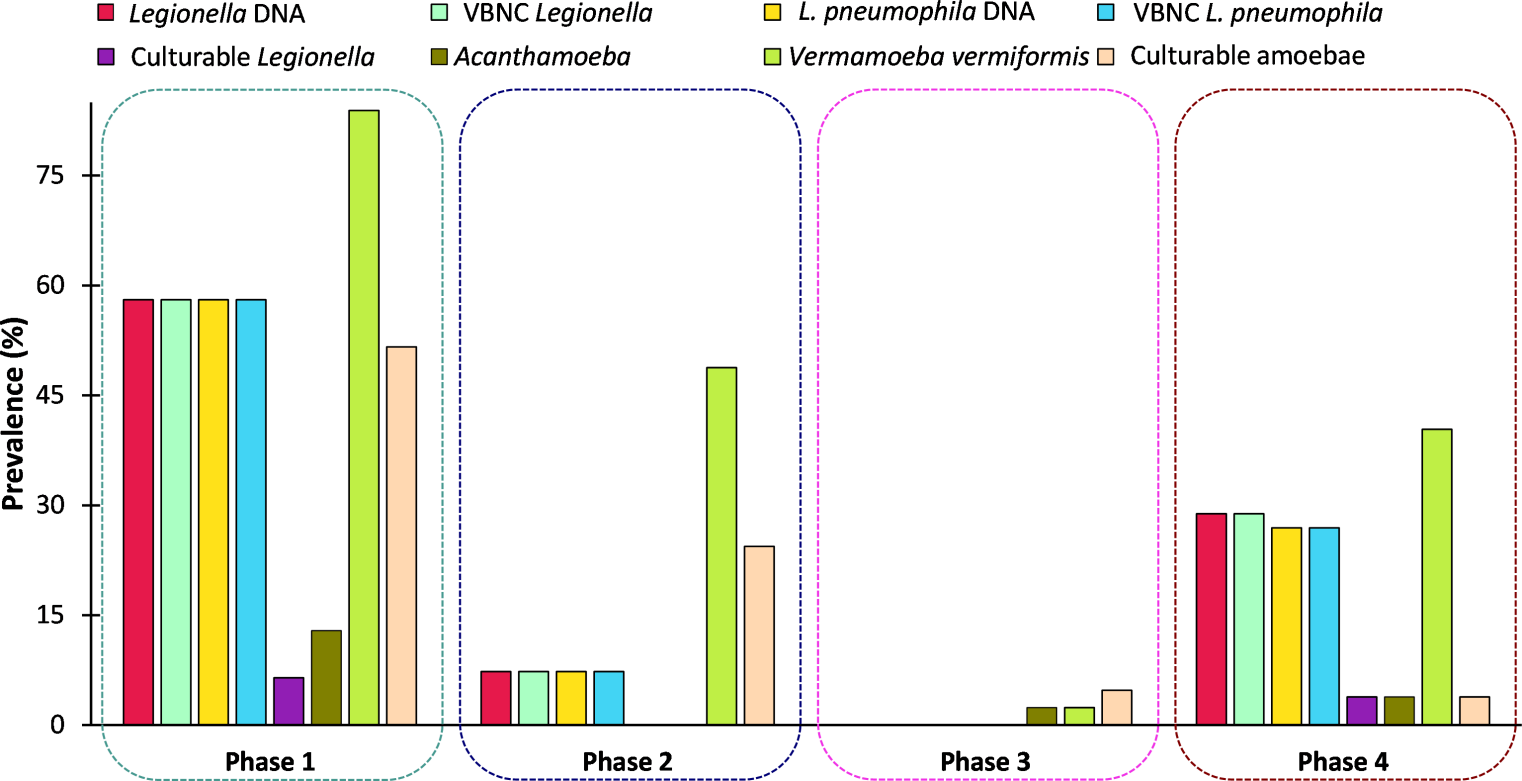
Relationship between intermittent water usage and the presence of *Legionella* spp./*Vermamoeba vermiformis*. X-axis represents total duration (hours) of flushing events recorded for one-month prior to sampling. Flushing was categorized into low; 0 to < 2 hours, and high flow regime; ≥ 2 to 40 hours. Y-axis represents log_10_(GU/L) of *Legionella* spp., VBNC *Legionella* spp., *L. pneumophila*, VBNC *L. pneumophila*, and *V. vermiformis*.

The total duration (hours) of flushing events for one month prior to sampling, was categorized into: low (0 to < 2 hours/month) and high (≥ 2 to 40 hours/month) flow regimes. The Kruskal-Wallis analysis indicated that the concentrations of *Legionella* spp. DNA (*p* < 0.01), *L. pneumophila* DNA (*p* < 0.01), VBNC *Legionella* spp. (*p* < 0.001), VBNC *L. pneumophila* (*p* < 0.001) and *V. vermiformis* DNA (*p* < 0.05), were all higher in low flow regimes compared with high flow regimes (**Table 3**; **Figure 3**). When the total duration (hours) of flushing events for only one week prior to sampling was examined, no association was observed with any of the microbial concentrations measured. The HPC load did not show any measurable difference in the low vs high flow regimes either one month or one week prior to sampling (**Table 3**). In contrast with the total flow duration, the total number of flow counts (number of flushing events) for either one week or one month prior to sampling was not associated with any significant change in any of the microbial concentrations measured. In conclusion, a month of reduced usage (< 2 hours water flushing per month) supports the proliferation of *Legionella* spp./*L. pneumophila* and *V. vermiformis* in hospital water system.

### 3.5 Influence of water temperature and on *Legionella* spp., HPC, and FLA

The water outlets (hand basins and showers) of the hospital water system received water from both the cold water supply and hot water supply (**Supplementary Material**; **Figures S3**, **S5**). The temperature data for each sample location were averaged over one week and one month prior to sample collection (**Supplementary Material**; **Figures S3**, **S5**). The average temperatures (mean ± SD) measured from the cold water supply were (21.78 ± 1.98°C per week and 22.01 ± 1.69°C per month), hot water supply (23.64 ± 3.15°C per week and 23.69 ± 3.08°C per month) and outlet water (23.74 ± 2.43°C per week and 23.76 ± 1.95°C per month). No relationships between microbial concentration and water temperatures were observed. This is likely due to the average water temperature being similar for both hot and cold water supplies, with increases in hot water temperature occurring through hot water usage having a limited effect on the overall average temperature due to the periods of stagnation and inactivation occurring in between usages (**Supplementary Material, Figures S4**, **S6**).

## 4 Discussion

In this study, it was identified that 31.3% (n = 21/67) hand basin water, 18.9% (n = 10/53) shower water and 10.9% (n = 5/46) biofilm samples were positive for either *Legionella* spp. or *L. pneumophila* gene marker (**Table 1**). According to the literature, the majority of engineered water systems of hospital and healthcare facilities are contaminated with *Legionella* spp. or *L. pneumophila*. In Poland, 74.7% of water samples from hospitals and other large building structures tested positive for *Legionella* spp., and *L. pneumophila* sg2-14 was the most prevalent serogroup (Sikora et al., 2015). A similar study conducted in Hungary that examined water samples from healthcare facilities and other buildings showed that 60% samples were positive for *Legionella* spp. (predominantly *L. pneumophila* sg2-14) (Barna et al., 2016). A study conducted in 20 different hospitals in Spain reported that 37.2% of water samples were colonized with *L. pneumophila* sg1 and *L. pneumophila* sg2-14 (Sabrià et al., 2004). In Taiwan, 63% of samples collected from hospital water systems tested positive for *Legionella* spp. and *L. pneumophila* sg1 (Yu et al., 2008). Comprehensive national surveillance studies conducted in 13 different states of the USA reported that 70% of hospital water systems were contaminated with *Legionella* spp. (Stout et al., 2007). A recent study conducted in Australia detected 41% samples of water and biofilms from hospital and residential buildings were colonized with *Legionella* spp. (Nisar et al., 2022). The lower *Legionella* spp. prevalence in this study could be due to the fact this was a case study of a single hospital that has been proactive in their water quality risk management compared with other hospitals.

All previous studies on *Legionella* spp. in engineered water systems have either used standard culturing or a qPCR assay to detect *Legionella* spp, and none have screened for the presence of VBNC *Legionella* spp. In the present study, VFC+qPCR assay showed that all water and biofilm samples positive for *Legionella* spp./*L. pneumophila* gene marker also contained VBNC cells. The quantity of total *Legionella* spp. detected by qPCR assay was greater than VBNC cells, which clearly highlights that the hospital water system harbored both dead and VBNC *Legionella* spp. (**Figure 1**). Our findings suggest that the standard *Legionella* spp./*L. pneumophila* guidelines should include quantification of VBNC cells.

Currently, there is still much debate around the exact infective dose for *Legionella* spp. (Bartram et al., 2007). An analysis by Sikora et al. (2015) estimated that legionellosis outbreaks may occur sporadically when water is contaminated with 10^3^ to 10^5^ CFU/L and when *Legionella* spp. counts exceed 10^5^ CFU/L an outbreak of legionellosis can occur (Sikora et al., 2015). However, these estimates are based on the number of culturable cells (CFU/L), so it is challenging to determine the relative risk associated with the concentrations of VBNC cells (GU/L). In contrast with culturable *Legionella* spp., VBNC cells infect with lower pathogenicity and take a longer time to infect amoebae (Nisar et al., 2023). Therefore, future research is needed to determine the infectious dose of VBNC *Legionella* spp. to understand the role of VBNC *Legionella* spp. in nosocomial infections and the public health risk posed by this concentration of VBNC *Legionella* spp. in engineered water systems.

In hospital water systems, *Legionella* infects and survives within protozoan hosts, including *Acanthamoeba* and *V. vermiformis* (Nisar et al., 2020a). In this study, it was identified that *V. vermiformis* (gene marker and culturable) was the most commonly identified amoebae associated with *Legionella* spp. prevalent in the water and biofilm samples (**Table 4**). This is supported by previous studies that have demonstrated *V. vermiformis* to be widely present in potable water (Kuiper et al., 2006; Nisar et al., 2020a). Similarly, microbiome analysis of potable water also showed *V. vermiformis* as the most prevalent protozoa (Delafont et al., 2013; Delafont et al., 2016). Water samples of dental units of Italian hospitals were found to be highly contaminated with *V. vermiformis* (60%) (Spagnolo et al., 2019). Similarly, a study conducted in hospitals of South Africa identified that 69% of samples were positive for *V. vermiformis* and 30.6% for *Acanthamoeba* (Muchesa et al., 2018). A recent study conducted in Australia examined water and biofilm samples from hospital and residential buildings showed the presence of FLA in 69% of the samples. It was also found that in all tested samples, *V. vermiformis* (55%) was the more frequently detected FLA (Nisar et al., 2022). In comparison with *Acanthamoeba*, *V. vermiformis* is more sensitive to disinfection treatments (Nisar et al., 2020a). Therefore, high levels of *V. vermiformis* could be attributed to decay and lower levels of residual chemical disinfectants in the hospital water system.

To our knowledge HPC loads have not been linked to any known legionellosis outbreak and the relationship between HPC levels and opportunistic premise plumbing pathogens is still unclear (Bartram et al., 2003). The SA Health, (2013) use HPC load as an indicator of water quality and recommend that if HPC load is ≥ 10^2^ CFU/mL in warm water systems then the disinfection procedures for engineered water system should be considered. In this study it was found that the water samples with high HPC loads (≥ 5 × 10^3^ CFU/L) contained high quantities of both *Legionella* spp. and *L. pneumophila* (**Figure 2**; **Table 3**). These results are in accordance with a previous study conducted on engineered water systems of residential buildings, which also showed a positive relationship between HPC levels and concentrations of *Legionella* spp. (Ley et al., 2020). Similarly, it was also found that samples with high HPC loads harbored high levels of *V. vermiformis* (**Figure 2**; **Table 3**). The relationship between bacteria and FLA consists of three major types of interactions i.e., mutualism, parasitism, and predation (Shi et al., 2021). Generally, FLA are considered natural predators of bacteria, which could account for the high levels of *V. vermiformis* observed in the presence of high levels of HPC (Rodriguez-Zaragoza et al., 2005). It was also identified that all samples positive for *Legionella* spp./*L. pneumophila* were also positive for FLA. Furthermore, Spearman’s analysis demonstrated a strong positive correlation between *Legionella* spp./*L. pneumophila* and *V. vermiformis*. In engineered water systems, FLA exist in both trophozoite (metabolically active) and cyst (dormant) states (Zhang and Lu, 2021). The trophozoites support intracellular proliferation of *Legionella* spp. and transformation of VBNC *Legionella* spp. into a culturable state (Solomon et al., 2000; Shadrach et al., 2005; Berk et al., 2008; Watanabe et al., 2016; Boamah et al., 2017). Amoebae cysts protect intracellular *Legionella* spp. from prolonged chemical and physical disinfection treatments (Dobrowsky et al., 2016; Boamah et al., 2017). The significant role amoebae play in *Legionella* spp. survival in potable water systems suggests that guidelines for the control of *Legionella* spp. must consider acceptable limits of amoeba within these systems as a measure to control *Legionella* spp. concentrations.

Water stagnation within building distribution systems promotes the accumulation of biomass, decay of chemical disinfectants, and alters the water quality (Bedard et al., 2018). Therefore, this study investigated the effect of temporary stagnation induced by intermittent flushing and water usage on *Legionella* spp., with a special focus on VBNC *Legionella* spp. It was found that an increase in temporary stagnation once a month prior to sampling significantly (*p* < 0.01) increased the quantity of total *Legionella* spp./*L. pneumophila* and VBNC *Legionella* spp./*L. pneumophila* population; however, increased stagnation one week prior to sampling was not associated with increased risk (**Figure 3**; **Table 3**). This supports guidelines that recommend routine flushing of outlets to manage *Legionella* spp. within engineered water systems (Enhealth, 2015). To our knowledge, this is first study in which the effect of temporary stagnation, HPC load, and *V. vermiformis* on VBNC *Legionella* spp./*L. pneumophila* in hospital water systems has been investigated. This study averaged water temperatures across one week or one month prior to sampling for both the hot and cold water pipelines/outlets. As a result water temperatures were more similar to each other than anticipated. This is likely to explain the lack of a statistically significant difference in *Legionella* concentrations associated with different temperatures. Future research with a larger dataset is needed to explore the temperature relationship further.

## 5 Conclusion

In building plumbing systems, temporary stagnation arising through intermittent usage causes water quality to deteriorate. This study identified that temporary stagnation for over a month promotes the persistence of VBNC *Legionella* spp./*L. pneumophila*. Similarly, FLA and heterotrophic bacteria present in this temporary stagnant environment positively interact with *Legionella* spp./*L. pneumophila*. Therefore, temporary stagnation, FLA and heterotrophic bacteria must be managed for the proper control and prevention of LD. This study also showed that the standard microbiological culture method used to detection *Legionella* spp. returned a false negative result for 88% of the VBNC *Legionella* spp. positive samples. As all samples positive for VBNC *Legionella* spp. were also qPCR positive, this suggests that qPCR may be a more appropriate detection method for routine surveillance. However, future research is needed to investigate the concentrations of VBNC *Legionella* spp. that pose a risk to public health to enable interpretation of these results to inform improved *Legionella* spp. guidelines.

## Data availability statement

The original contributions presented in the study are included in the article/**Supplementary Material**. Further inquiries can be directed to the corresponding author.

## Author contributions

MN and HW conceived and designed research. MN performed the experiments. GB assisted in the flow cytometry. KR, MB, HW and RB provided technical assistance. JX and JH assisted in sampling and data collection. MN and HW drafted and edited the manuscript. HW, KR, MB, RB, GB, JX, and JH corrected and contributed to the manuscript. All authors approved of the final manuscript.
