# Supplementary material for: Stagnation arising through intermittent usage is associated with increased viable but non culturable Legionella and amoeba hosts in a hospital water system
Source: Front Cell Infect Microbiol. 2023 Jun 7;13:1190631. doi: 10.3389/fcimb.2023.1190631 (PMC10282743; doi:10.3389/fcimb.2023.1190631)
Supplement: Supplementary file 1 [file DataSheet_1.docx]

Supplementary Material

Water stagnation arising through intermittent usage is associated with increased viable but non culturable *Legionella* and amoeba hosts in a hospital water distribution system

Muhammad Atif Nisar^1^, Kirstin E. Ross^1^, Melissa H. Brown^1,2^, Richard Bentham^1^, Giles Best^3,4^, James Xi^5^, Jason Hinds^5^, and Harriet Whiley^1, 2^

^1^College of Science and Engineering, Flinders University, Bedford Park, SA, Australia

^2^ARC Training Centre for Biofilm Research and Innovation, Flinders University, Bedford Park, SA, Australia

^3^College of Medicine and Public Health, Flinders University, Bedford Park, SA, Australia

^4^Flow Cytometry Facility, Flinders University, Bedford Park, SA, Australia

^5^Enware Australia Pty Ltd, Caringbah, NSW, Australia

*** Correspondence:**

Corresponding Author

Harriet.Whiley@flinders.edu.au

# Supplementary Figures and Tables

**Table S1:** Sequences of oligos and fluorogenic probes used for qPCR assays.

| Oligo name | Sequence and fluorogenic signal (5´→3´) | qPCR assay conditions |
| --- | --- | --- |
| *Legionella* 16S rDNA gene specific qPCR primers and probe (International Organization for Standardization, 2019) | | |
| Forward oligo | GGAGGGTTGATAGGTTAAGAGCT | Step-I: one cycle of 95°C/3 min  Step-II: 43 cycles of 95°C/20 s and 60°C/60 s |
| Reverse oligo | CCAACAGCTAGTTGACATCGTTT |  |
| Probe* | FAM–AGTGGCGAAGGCGGCTACCT–Q |  |
| *L. pneumophila mip* gene specific qPCR primers and probe (International Organization for Standardization, 2019) | | |
| Forward oligo | CCGATGCCACATCATTAGC | Step-I: one cycle of 95°C/3 min  Step-II: 43 cycles of 95°C/20 s and 60°C/60 s |
| Reverse oligo | CCAATTGAGCGCCACTCATAG |  |
| Probe* | FAM–TGCCTTTAGCCATTGCTTCCG–Q |  |
| *Acanthamoeba* 18S rDNA gene specific qPCR primers and probe (Qvarnstrom et al., 2006) | | |
| Forward oligo | CCCAGATCGTTTACCGTGAA | Step-I: one cycle of 95°C/3 min  Step-II: 40 cycles of 95°C/20 s and 63°C/60 s |
| Reverse oligo | TAAATATTAATGCCCCCAACTATCC |  |
| Probe* | FAM–CTGCCACCGAATACATTAGCATGG–Q |  |
| *Vermamoeba vermiformis* 18S rDNA gene specific qPCR primers and probe (Scheikl et al., 2016) | | |
| Forward oligo | TAACGATTGGAGGGCAAGTC | Step-I: one cycle of 95°C/5 min  Step-II: 45 cycles of 95°C/20 s and 60°C/60 s |
| Reverse oligo | ACGCCTGCTTTGAACACTCT |  |
| Probe* | FAM–TGGGGAATCAACCGCTAGGA–Q |  |

*FAM: 6-carboxyfluorescein λ_(excitation)_/λ_(emission)_ 495/520 nm, channel for qPCR: λ_(source)_ 470 nm and λ_(detector)_ 510 nm; Q: Iowa Black^®^ FQ quencher with absorbance spectrum range λ 420 nm to 620 nm with λ_(max)_ 531 nm; s: seconds; min: minutes

**References**

International Organization for Standardization (2019). "ISO/TS12869:2019 Water quality - Detection and quantification of *Legionella* spp. and/or *Legionella pneumophila* by concentration and genic amplification by quantitative polymerase chain reaction (qPCR)".).

Qvarnstrom, Y., Visvesvara, G.S., Sriram, R., and Da Silva, A.J. (2006). Multiplex real-time PCR assay for simultaneous detection of *Acanthamoeba* spp., *Balamuthia mandrillaris*, and *Naegleria fowleri*. *J Clin Microbiol* 44**,** 3589-3595.

Scheikl, U., Tsao, H.F., Horn, M., Indra, A., and Walochnik, J. (2016). Free-living amoebae and their associated bacteria in Austrian cooling towers: A 1-year routine screening. *Parasitol Res* 115**,** 3365-3374.


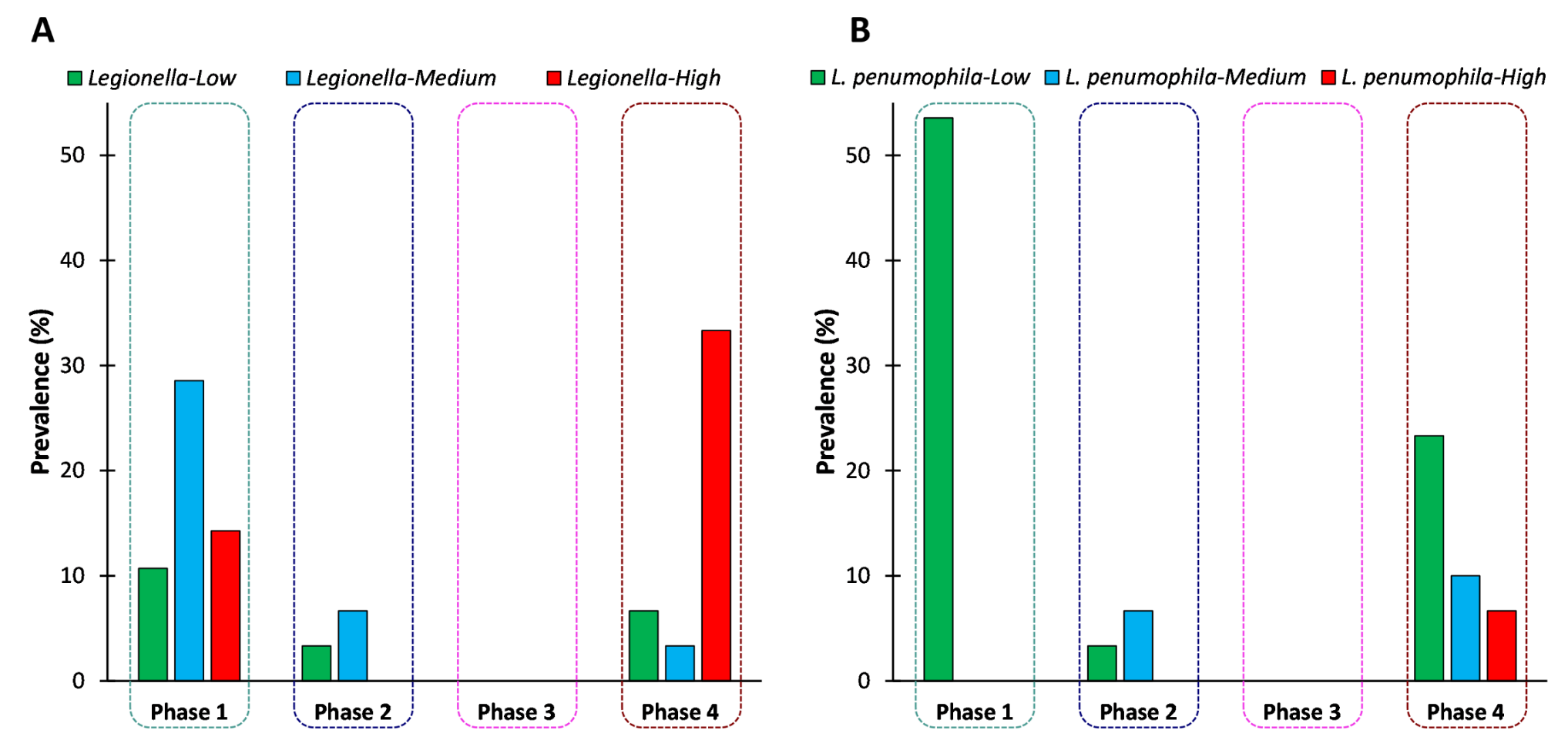


**Figure S1:** The percentage of water samples tested for VBNC *Legionella* spp. (A) and VBNC *L. pneumophila* (B) with three levels of contamination. The amount of VBNC cells were divided into three levels: low (< 10^3^ GU/L, green), medium (10^3^ to 10^4^ GU/L, blue) and high (> 10^4^ GU/L, red) contamination. Total of 120 water samples (hand basin and shower) were collected in March 2021 (Phase 1), April 2021 (Phase 2), November 2021 (Phase 3), and June 2022 (Phase 4). Y-axis represents % positive samples and X-axis represents sampling phases.


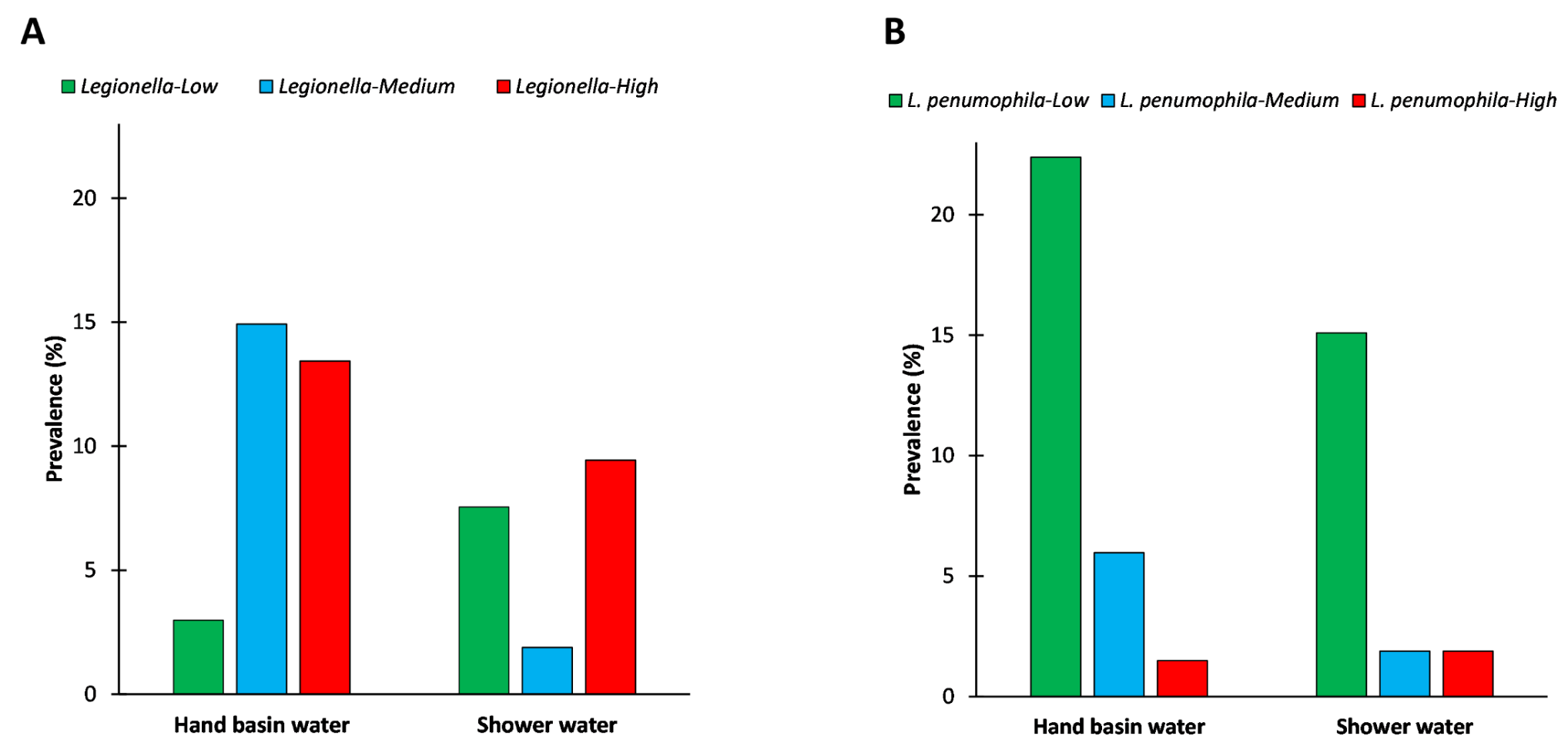


**Figure S2:** The percentage of water samples tested for VBNC *Legionella* spp. (A) and VBNC *L. pneumophila* (B) with three levels of contamination in hand basin (n = 67) and shower water (n = 53) samples. The amount of VBNC cells were divided into three levels: low (< 10^3^ GU/L, green), medium (10^3^ to 10^4^ GU/L, blue) and high (> 10^4^ GU/L, red) contamination. Y-axis represents % positive samples and X-axis represents sample type.

**
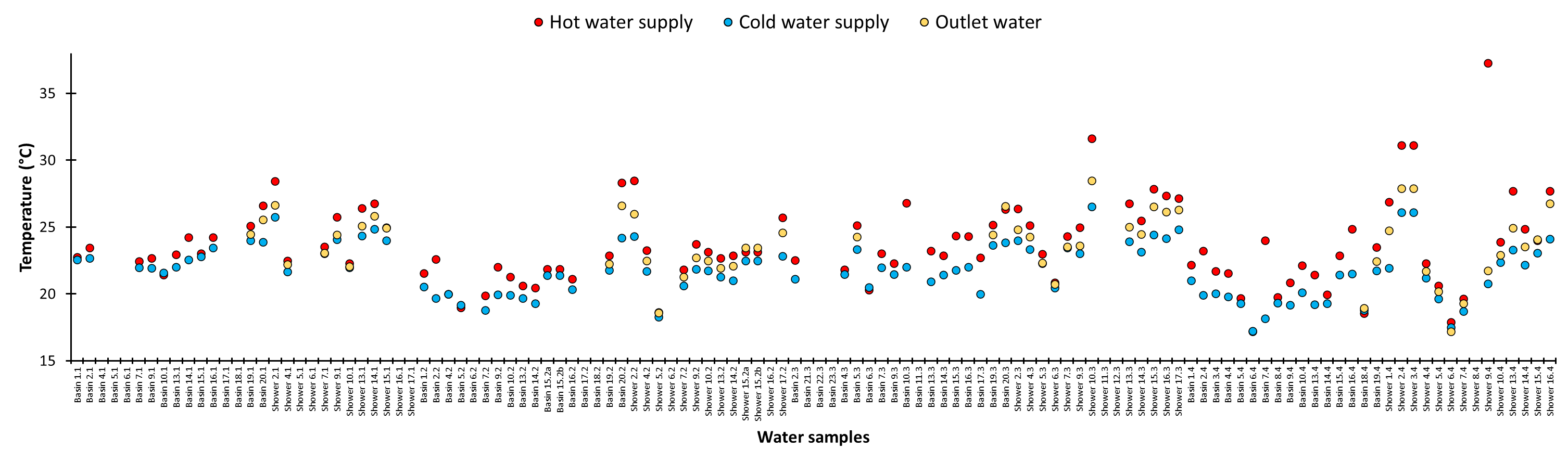
**

**Figure S3:** Average temperature of hot water supply, cold water supply and outlet water (hand basin and shower) recorded for one-week prior to sampling. X-axis represents the tested water samples and y-axis represents temperature (°C). Y-axis represents temperature of water one-week prior sampling (cold water supply, hot water supply and outlet water) and X-axis represents the samples.


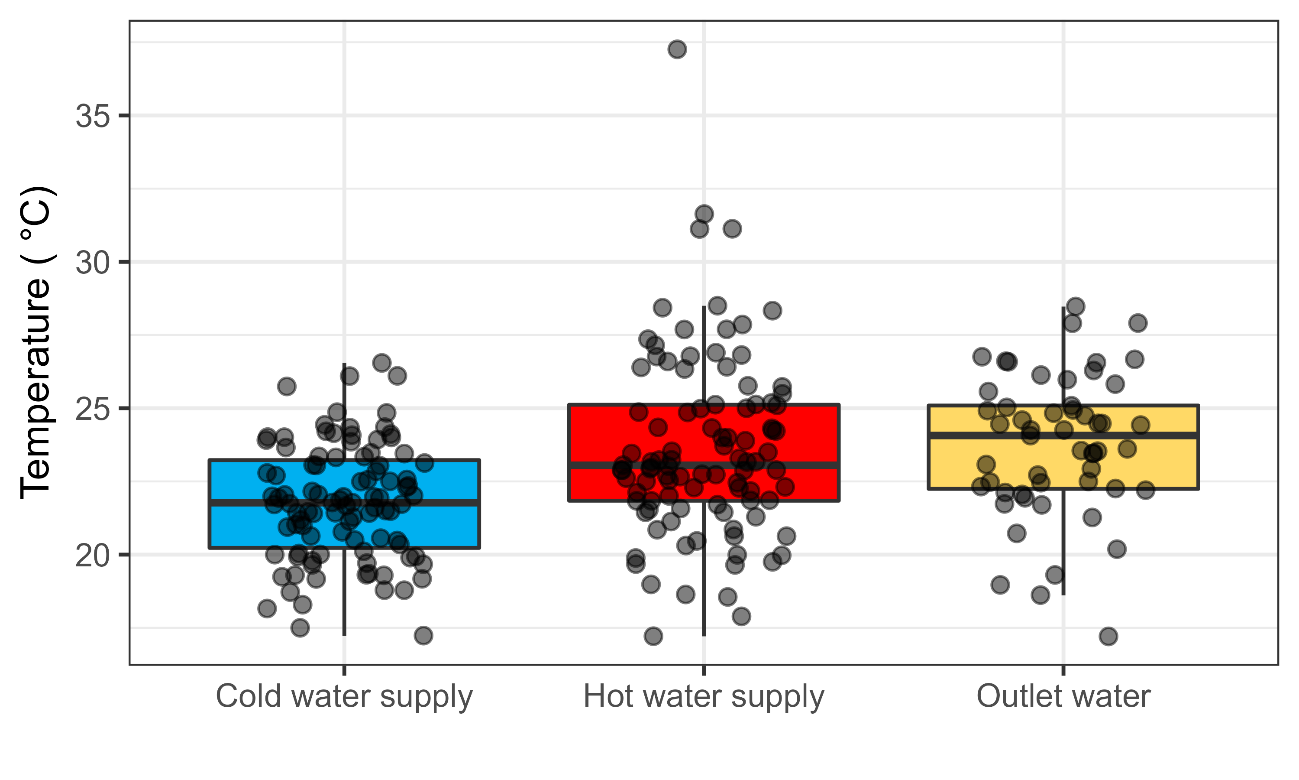


**Figure S4:** Average temperature of hot water supply, cold water supply and outlet water (hand basin and shower) recorded for one-week prior to sampling. Y-axis represents temperature of water one-week prior sampling and X-axis represents the samples.

**
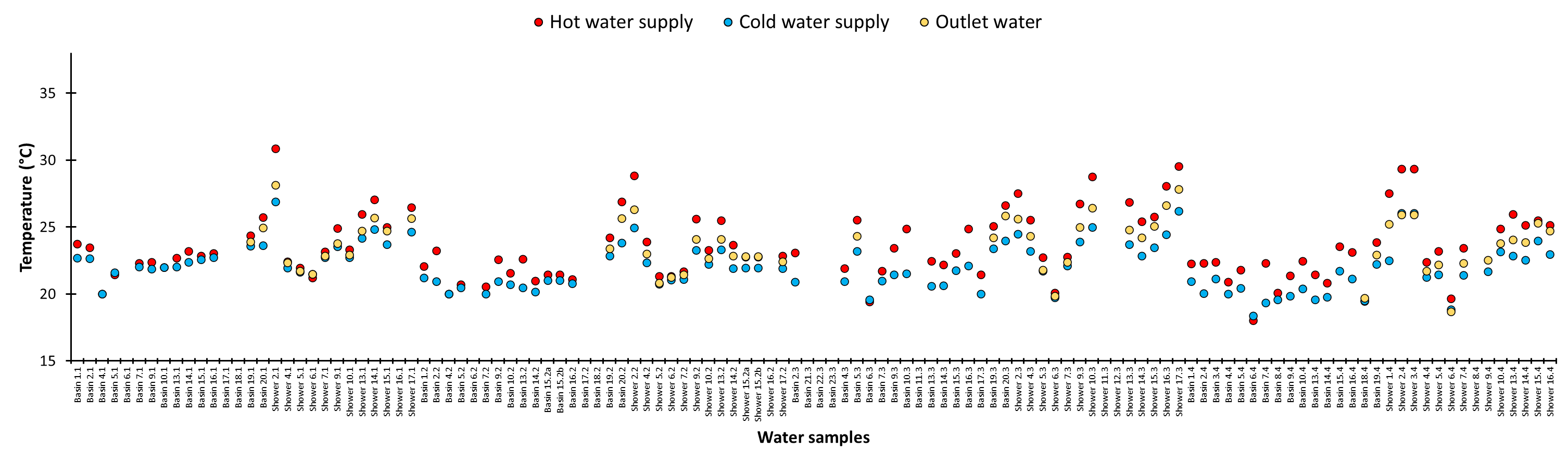
**

**Figure S5:** Average temperature of hot water supply, cold water supply and outlet water (hand basin and shower) recorded for one-month prior to sampling. X-axis represents the tested water samples and y-axis represents temperature (°C).


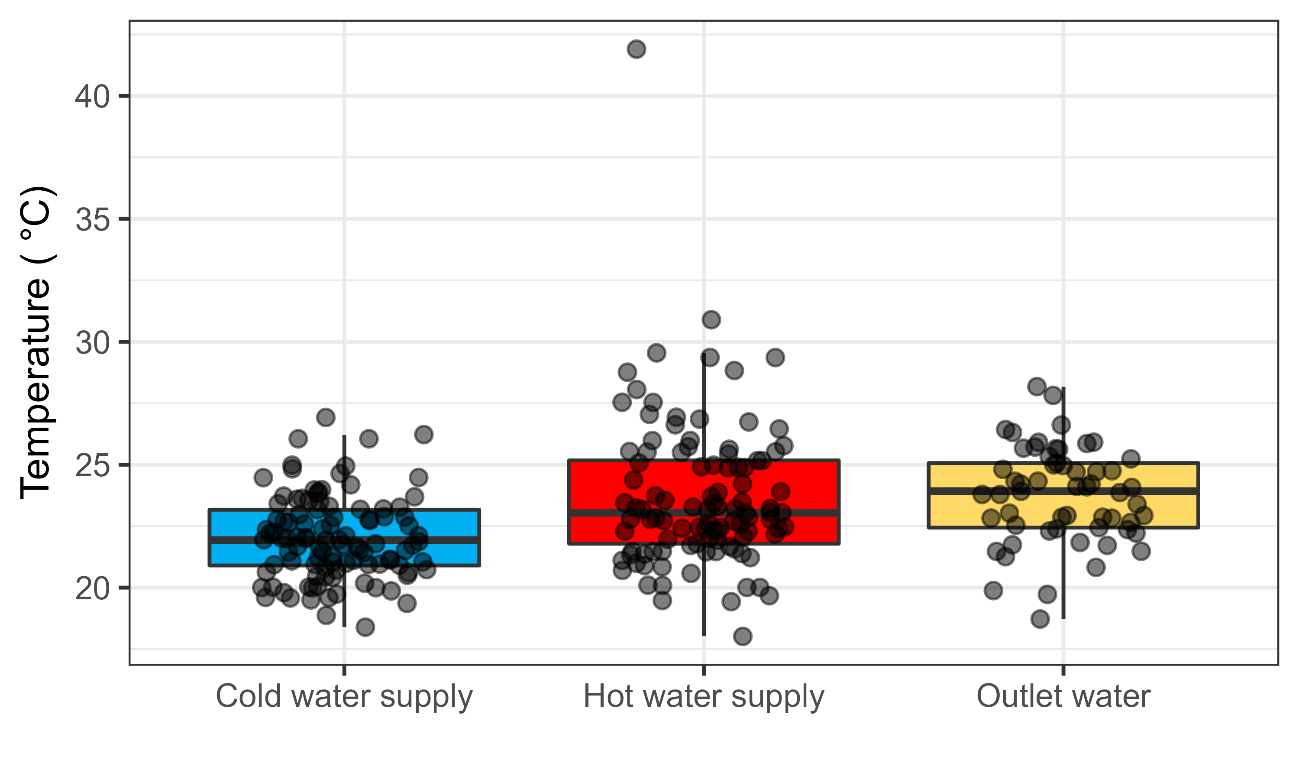


**Figure S6:** Average temperature of hot water supply, cold water supply and outlet water (hand basin and shower) recorded for one-month prior to sampling. Y-axis represents temperature of water one-month prior sampling (cold water supply, hot water supply and outlet water) and X-axis represents the samples.
